# Service providers’ self-perceived competence in supporting women with disabilities subjected to intimate partner violence: insights from a Swedish survey

**DOI:** 10.1080/16549716.2025.2476822

**Published:** 2025-04-07

**Authors:** Cartrine Anyango, Erling Häggström Gunfridsson, Fredinah Namatovu

**Affiliations:** a Department of Epidemiology and Global Health, Umeå University, Umeå, Sweden; b Centre for Demographic and Ageing Research (CEDAR) Umeå University, Umeå Sweden

**Keywords:** Intimate partner violence, women with disabilities, formal support, self-perceived competence, service providers

## Abstract

**Background:**

Intimate partner violence (IPV) is a global issue, with women, especially those with disabilities, facing a higher lifetime risk than those without disabilities. Given the elevated risk factors and challenges related to having a disability, it is crucial to provide effective IPV support. The competence and expertise of service providers regarding IPV significantly influence their ability to provide adequate IPV support. Understanding service providers’ self-perceived competence is essential for improving the quality of IPV support for women with disabilities.

**Objective:**

This study assesses the self-perceived competence of service providers in supporting women with disabilities subjected to IPV in Sweden.

**Methods:**

A cross-sectional survey was distributed to professionals in healthcare, social services, and the police, and 1,151 people participated. Descriptive statistics and linear regression analyses were performed to assess the factors influencing service providers’ self-perceived competence.

**Results:**

The findings indicate that healthcare, police, and social services professionals often encounter women with disabilities, but they rarely ask them directly about IPV. Many don’t routinely inquire about IPV exposure. While institutional routines for addressing IPV exist, service providers don’t consistently implement or use them. Key factors influencing self-perceived competence include receiving IPV and disability-specific training and sufficient employer support for addressing IPV among women with disabilities.

**Conclusions:**

The findings underscore the need for a more consistent application of routines and enhanced training to strengthen the capacity of service providers to support women with disabilities subjected to IPV.

## PAPER CONTEXT

- **Main findings:** Our study highlights broader systemic gaps in how IPV is addressed within the context of disability, signaling the need for tailored support strategies and further research to ensure that disabled women subjected to IPV receive the comprehensive support they need.
- **Added knowledge:** The underutilization of established routines for disabled victims of IPV suggests a need for training, and the enforcement of these routines within support institutions.
- **Global health impact for policy and action:** Our findings have important implications for policy, practice, and future research. Formal support institutions need to improve routine IPV inquiry processes to ensure that women with disabilities are routinely asked whether they have been subjected to IPV.

## Background

Intimate partner violence (IPV) remains a pervasive global issue, with significant implications for the well-being of affected women. Approximately 30% of ever-partnered women have experienced IPV during their lifetime [1]. In Europe, available statistical estimates suggest that 8% of women have experienced physical violence, 32% psychological abuse, and 5% financial abuse [2]. IPV comprises physical, sexual, or psychological harm perpetrated by a current or former intimate partner [3]. Although there have been concerted efforts to address IPV, it continues to disproportionately affect women in general, and even more those in vulnerable groups such as women with disabilities [4,5]. A recent systematic review, which included 26 studies, found that women with disabilities face a higher frequency and risk of IPV than women without disabilities [6]. Women with disabilities are vulnerable to multiple and repeated acts of violence and abuse due to factors such as stigmatization, reliance on the perpetrators, poverty, and social isolation [6–8].

Due to the elevated risk of becoming victims of IPV, it is important to provide effective formal IPV support for women with disabilities. Such support requires, among other things, service providers with the necessary skills and knowledge [9]. Nonetheless, IPV support for women with disabilities is often of poor quality and insufficiently resourced [10]. Research indicates that, while women with disabilities experience IPV at higher rates, their access to IPV support and the effectiveness of such support remains limited compared to that available to other women [11].

Service providers’ competence and knowledge about IPV can significantly influence their ability to provide adequate support [12]. Competent service providers need to be able to ask about IPV (routine IPV inquiry), identify IPV signs, provide tailored support, and facilitate appropriate referrals to other support avenues for further targeted support. Yet reports from various European regions indicate inadequacy in service providers’ competence regarding disability and gender-based violence, which hinders effective support [3,13].

Our study, conducted in the context of Sweden, considers IPV support to consist of a range of assistance, services, protection, safety, and care offered by professionals working in formal support institutions. In Sweden, directives from the National Board of Health and Welfare (NBHW) emphasize that professionals conducting interventions under the Social Services Act (2001:453) should be knowledgeable about violence and other forms of abuse, able to identify victims, and able to ensure they receive adequate support. Moreover, such personnel are expected to apply their knowledge and skills in practical situations to support women exposed to IPV [13]. Also, the law regarding support for people with disabilities emphasizes that their support should be well coordinated, tailored to their needs, and easy to access. This is unique to Sweden, as these services are provided within the welfare system and are free for everyone to access. Thus, in Sweden, the institutions that provide support for women exposed to IPV include healthcare, the police, women’s shelters operated by feminist organizations, and social services. Although such diverse formal support institutions exist, research indicates suboptimal use of IPV support within those institutions [14,15].

### IPV and disability-competent professionals

Professional competence refers to the proficient application of knowledge and skills within a specific professional setting [16]. The translation of theoretical knowledge and skills into practical competencies is often shaped by both the professional environment and individual capabilities [16]. Self-efficacy or confidence, such as belief in one’s abilities, skills, and capability to execute a specific task, can influence one’s decision-making and persistence in pursuing set objectives within a given context [17]. Previous research indicated that social workers exhibit high self-efficacy in general social work but lower confidence in tasks specifically targeted towards IPV victims [18].

To effectively support people with disabilities, professionals need to be competent in addressing both disability and IPV. Literature shows that a disability-competent workforce is necessary for delivering high-quality support to people with disabilities [19]. Disability-competent service providers are equipped to offer tailored support, conduct appropriate inquiries, provide timely follow-ups, and respond adequately to the unique needs of people with disabilities [19]. Training service providers is essential in order to enhance their understanding and methods of supporting people with disabilities [20]. Additionally, research has shown that training on IPV significantly enhances nurses’ confidence and comfort when working with IPV victims [21]. In Sweden, both professional experience and IPV focused training are associated with greater perceived competence and a greater likelihood of inquiring about abuse [18]. Competent providers are also more likely to regularly screen for IPV [22].

Despite the recognized importance of service providers possessing competence in addressing IPV among the disabled, and the existing gaps in research, there is a notable lack of studies in Sweden examining service providers’ self-perceived competence in providing IPV support to women with disabilities. Available evidence from the social services setting shows that a significant proportion of social workers perceive themselves as inadequately prepared to handle IPV cases [22]. If we are to improve the quality and effectiveness of IPV support for women with disabilities, it is crucial to understand service providers’ self-perceptions because providers’ self-perceived competence directly influences their ability and confidence to deliver appropriate support. By identifying and addressing gaps in these self-perceptions, targeted improvements can enhance the support provided to women with disabilities.

Thus, the aim of this study was to assess service providers’ self-perceived professional competence in providing IPV support to women with disabilities in Sweden.

## Methods

### Study design

This study adopted a cross-sectional design using data collected via a questionnaire. The development of the questionnaire was a dynamic and cooperative process, engaging both the research team and Statistics Sweden (SCB). SCB is a government agency responsible for producing the official statistics in Sweden. The final questionnaire underwent a pilot test. We selected a few colleagues, who are researchers but not part of this research team, to respond to the questionnaire, evaluating its flow, ease of use, and question clarity, and they provided useful feedback that further improved the final instrument.

### Study setting and IPV support in Sweden

In Sweden, the National Board of Health and Welfare (NBHW) plays a pivotal role in ensuring equitable access to healthcare, social welfare, and quality services for all the country’s residents, including those with disabilities. The NBHW emphasizes the same level of care and support as their non-disabled counterparts [13]. Sweden’s disability policy framework prioritizes the full integration of individuals with disabilities into society, emphasizing participation and equality [23]. Consequently, service providers in Sweden are expected to adhere to principles that address the unique needs of individuals who have experienced violence, considering factors such as their age, disability status, sexual orientation, and substance use or addiction [13]. To begin with, according to NBHW [13], the support institutions have information about IPV on their websites and operate helplines for those in need of IPV support or who want to report violence.

Specifically, the healthcare sector focuses on the early detection and prevention of IPV. This approach involves integrating routine IPV inquiries into healthcare procedures. The police sector recognizes IPV as a global issue and operates within the Swedish criminal justice system [24]. Each police district maintains specialized units that utilize structured violence risk assessment tools. These tools inform case prioritization and guide risk management measures, including safety discussions, collaboration with social services, and access to shelters [25]. In the social services sector, IPV work is implemented across all 290 municipalities in Sweden. Local Social Welfare Committees steer such work, guided by the Swedish Social Services Act [13].

### Study population and sampling procedure

This study’s participants were service providers across specific professional groups: healthcare, social services, and the police. To create a sample, data from the 2020 occupational register was used to define and identify potential study participants within the population. Initially, there were 152,250 individuals in the pool. Before drawing the sample, this group was cross-checked against the latest population registry to eliminate any overlap – primarily individuals who had passed away or emigrated. The sampling frame was then stratified based on occupational code, resulting in 13 distinct strata:(1) Doctors, (2) Basic trained nurses, (3) Midwives, (4) District nurses, (5) Psychiatric nurses, (6) Geriatric nurses, (7) Other specialist nurses, (8) Psychologists, (9) Psychotherapists, (10) Social workers, (11) Counselors, (12) Needs assessors, and (13) Police officers. From this frame, a stratified random sample of 3,500 individuals was drawn. In a stratified random sample, all subjects within a stratum have an equal chance of inclusion. Accounting for eight overlapping individuals, the final sample size was 3,492 individuals.

### Data collection

The data collection was conducted by SCB on behalf of Umeå University. The survey was conducted between September and November 2022 and took approximately 20 minutes to complete. Initially developed in both Swedish and English, the final questionnaire distributed to the study participants was in Swedish. It was comprehensive and consisted of 38 questions. The questions were categorized into those related to the respondents’ work and experience, their interactions with people with disabilities, and specifically those who have been subjected to IPV; the support provided by the employer, routines and guidelines, training on IPV and disability and questions on self-perceived competency – which are used in this study. To engage respondents, four separate mailings were sent out via the post – the first containing the questionnaire, and the remaining three containing reminders. Each mailing included an introductory letter, requesting recipients to complete the questionnaire and return it to SCB. In total, 1,151 individuals responded, giving a 32.9% response rate.

### Variables

#### The outcome variable

Self-perceived competence was assessed through five questions that gauged the participants’ confidence in supporting people with disabilities exposed to IPV. To evaluate participants’ self-perceived competence, service providers were asked: ‘When it comes to people with disabilities who have experienced IPV, do you feel that you have the competence to: (1) routinely ask questions about IPV, (2) identify signs of IPV, (3) handle IPV, (4) provide support or intervention to prevent IPV, and (5) refer victims to other support services?’ Response options were: ‘Yes,’ ‘Yes, partly,’ and ‘No.’ We assigned scores to these responses: ‘Yes’ = 1, ‘Yes, partly’ = 0.5, and ‘No’ = 0. We calculated an average score from the five questions to determine the overall self-perceived competence score, resulting in a continuous outcome variable.

#### Demographic variables

These included gender, age group, country of origin, and education level. These were retrieved from the total population register. Additionally, other demographic variables collected through the questionnaire included occupation, years worked, and type of employment.

#### Availability and use of routines

These variables assessed whether respondents’ workplaces had routines and, if so, whether they used those routines. In our study, routines are defined as a set of structured actions, procedures, and guidelines designed to support IPV victims. The variables included the availability of ‘routines to support victims of IPV,’ and ‘routines to support people with disabilities exposed to IPV.’ Participants who responded that they had routines to support people with disabilities exposed to IPV were also asked how often they had used those routines in the last 12 months.

#### Training on disability and IPV

The variable ‘hours of training’ assessed how many hours of competence training participants had received in relation to providing IPV support for women with disabilities.

#### Sufficient employer support

The variable ‘sufficient employer support for women with disabilities’ assessed whether the respondents felt that their employers provided them with sufficient support for them to adequately provide IPV support for women with disabilities.

### Data analysis

We computed descriptive statistics to highlight the frequencies of the participants’ characteristics. We then conducted analysis of variances (ANOVA) for each categorical variable to test for significant differences in self-perceived competence across the various categories. Based on the results of the ANOVAs, significant variables were included in a multiple linear regression analysis.

To understand the factors influencing service providers’ self-perceived competence, we developed five linear regression models. Models 1 through 4 each included a single study variable while controlling for gender and occupation, thereby allowing us to assess the individual effects of these variables. Specifically, Model 1 assessed the effect of hours of training; Model 2 examined the impact of employer support; Model 3 analyzed the effect of routines for supporting IPV victims; and Model 4 evaluated the effect of routines specifically for disabled IPV victims. Model 5 incorporated all of the study variables concurrently. After the regression analysis, we further conducted post-regression ANOVAs to assess the overall model fit – to check whether the included variables remained significant within the models.

All statistical analyses were performed using Stata version 14.2. We report parameter estimates using *p* values and 95% confidence intervals. Additionally, we present *p* values from F tests for each variable, which were used in testing whether the inclusion of a given variable contributes significantly to the model beyond the effects of other variables.

### Ethical considerations

The study obtained ethical approval from the Swedish Ethical Review Authority [J. Reg no. 2019–05249].

An introductory letter specifying essential information about the study’s background and purpose was sent to the survey respondents. This letter clarified that the survey was a collaborative work between Umeå University and SCB. Participants were informed about data retrieval from SCB’s register and the subsequent provision of an anonymized data file to Umeå University. Additionally, the letter highlighted data protection regulations, confidentiality, and that participation was voluntary. This transparent communication ensured that participants were well informed and could provide informed consent regarding their participation in the survey.

## Results

Table 1 presents participants’ demographic characteristics. A total of 1,151 service providers from various support institutions in Sweden, including healthcare, social services, and the police, responded to the survey. The majority were women (81.3%) and predominantly born in Sweden (90%). Nearly half of the participants (48.4%) were aged between 45 and 64. The vast majority (96.5%) had completed education beyond upper secondary school or high school (gymnasium). The largest proportion (61.3%) worked in healthcare, and over half (52.6%) had more than ten years of experience. Most of the service providers (73.3%) were permanently employed.

**Table 1.** Participants’ demographic characteristics (*N* = 1151).

Variable	N (%)
**Gender**	
Male	215 (18.7)
Female	936 (81.3)
**Country of Birth**	
Sweden	1036 (90.0)
Outside Sweden	115 (10.0)
**Age-group**	
24–44	419 (36.4)
45–64	557 (48.4)
65+	175 (15.2)
**Occupation**	
Police	64 (5.6)
Social services	182 (15.8)
Healthcare	706 (61.3)
Others	198 (17.2)
Missing	1 (0.1)
**Years Worked (years of experience)**	
0–5 years	171 (14.9)
6–9 years	169 (14.7)
More than 10 years	605 (52.6)
Missing	206 (17.9)
**Type of Employment**	
Permanent Employee	844 (73.3)
Temporary employee	18 (1.6)
Project/Hourly employee	40 (3.5)
Others	46 (4.0)
Missing	203 (17.6)

## Meeting women with disabilities exposed to IPV

Table 2 outlines the service providers’ contact with women with disabilities exposed to IPV. In response to the question: ‘Have you met a person with a disability in the last 12 months at your workplace?’, 70.3% of the service providers responded ‘yes.’ Of those, about 46% had met women with disabilities exposed to IPV, while 6.2% had been in contact with women with disabilities exposed to IPV more than ten times during the previous 12 months.

**Table 2.** Service providers’ contact with women with disabilities exposed to IPV.

Met^1^ a person with disabilities during the last 12 months at workplace (*N*=1151)	N (%)
Yes	809 (70.3)
No	134 (11.6)
Missing	208 (18.1)
**Number of times met women with disabilities at workplace during the last 12 months (*N*=809)**	
1 time	64 (7.9)
2–5 times	224 (27.7)
6–10 times	83 (10.3)
More than 10 times	426 (52.7)
Missing	12 (1.5)
**Number of times met women with disabilities exposed to IPV at workplace during the last 12 months (*N*=809)**	
Never	426 (52.7)
1 time	117 (14.5)
2–5 times	164 (20.3)
6–10 times	40 (4.9)
More than 10 times	50 (6.2)
Missing	12 (1.5)

Met means a service provider had professional contact with women with disabilities, seeking services not limited to IPV.

## Asking about IPV

**Figure 1.**
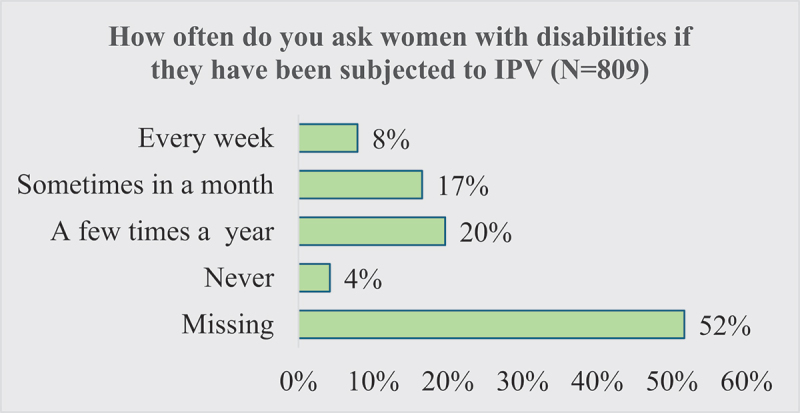
Asking about IPV.

Out of 809 service providers who had met women with disabilities during the previous 12 months at their workplace, as shown in Figure 1, only 8% asked about IPV weekly, 20% asked a few times a year, and 4% never asked. Notably, many of the service providers (52%) did not provide any response.

## Availability and use of routines

Regarding the availability of IPV support routines, Table 3 shows that 49.5% of the service providers reported that their workplace provides general support routines for people with disabilities, while 53.9% have support routines for IPV victims. Approximately 38% have specific routines to support people with disabilities exposed to IPV.

**Table 3.** Availability of routines *N* = 1151.

Routines	Yes	No	Missing
To support people with disabilities	570 (49.5%)	335 (29.1%)	246 (21.4%)
To support victims of IPV	620 (53.9%)	282 (24.5%)	249 (21.6%)
To support people with disabilities exposed to IPV	435 (37.8%)	450 (39.1%)	266 (23.1%)

**Figure 2.**
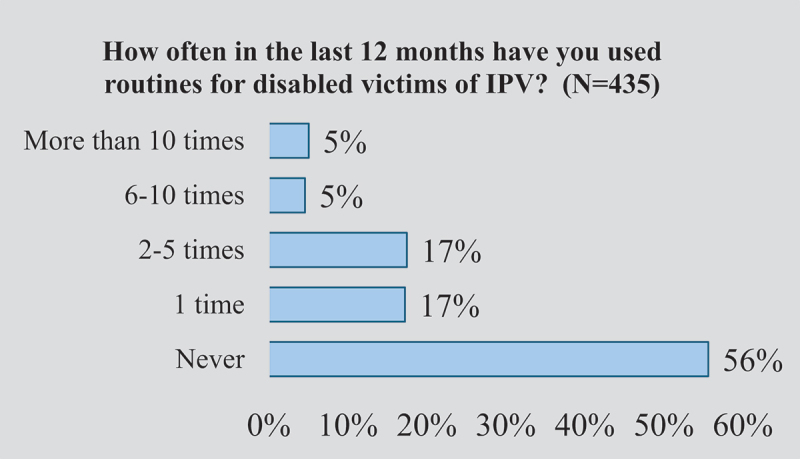
Use of routines.

Among the group that reported having routines to support people with disabilities exposed to IPV (*N* = 435), when asked about the frequency of utilizing these specific routines, as shown in Figure 2, 56% stated they had never used the routines during the previous 12 months while only 5% of service providers had used the routines more than 10 times during the previous 12 months.

## Multiple linear regression: factors influencing service providers’ self-perceived competence

The linear regression analysis results, detailed in Table 4, revealed the following:

**Table 4.** Multiple linear regression.

Variables/factors	Model 1: β(CI)	Model 2: β(CI)	Model 3: β(CI)	Model 4: β(CI)	Model 5: β(CI)
**Gender**	p value *0.002*	p value 0.155	p value 0.051	p value 0.082	p value 0.081
Female	Ref	Ref	Ref	Ref	Ref
Male	0.07**[0.02,0.11]	0.03 [−0.01,0.08]	0.04 [−0.00,0.09]	0.04 [−0.01,0.08]	0.04 [−0.00,0.08]
**Occupation**	p value 0.001	p value 0.000	p value 0.094	p value 0.000	p value e 0.003
Healthcare	Ref	Ref	Ref	Ref	Ref
Police	0.13***[0.06,0.20]	0.14*** [0.07,0.21]	0.07* [0.01,0.14]	0.15*** [0.08,0.22]	0.12***[0.06,0.19]
Social services	0.03 [−0.01,0.07]	0.08*** [0.04,0.12]	0.03 [−0.01,0.07]	0.06** [0.02,0.11]	0.00[−0.03,0.04]
Other	0.01 [−0.46,0.49]	0.22 [−0.26,0.70]	0.03 [−0.44,0.49]	0.19 [−0.28,0.67]	0.07 [−0.37,0.51]
**Hours of training related to people with disabilities victims of IPV**	p value 0.000				p value 0.000
None	Ref				Ref
1–5 hours	0.17***[0.13,0.21]				0.11***[0.08,0.15]
6–10 hours	0.18***[0.11,0.25]				0.11** [0.04,0.18]
11–20 hours	0.27***[0.18,0.36]				0.20***[0.11,0.29]
20 hours or more	0.34***[0.27,0.41]				0.26*** [0.20,0.33]
**Sufficient employer support for women with disabilities**		p value 0.000			p value 0.000
No		Ref			Ref
Yes		0.20*** [0.16,0.25]			0.12*** [0.08,0.17]
Partly		0.13*** [0.09,0.17]			0.07*** [0.03,0.11]
**Routines to support IPV Victims**			p value 0.000		p value 0.000
No			Ref		Ref
Yes			0.22***[0.19,0.26]		0.14*** [0.10,0.18]
**Routines to support people with disabilities exposed to IPV**				p value 0.000	p value 0.403
No				Ref	Ref
Yes				0.18***[0.15,0.21]	0.02 [−0.02,0.06]
**Adjusted R**^**2**^	0.18	0.11	0.18	0.16	0.30

β: Coefficient.

CI: 95% confidence intervals.

* *p* < 0.05, ** *p* < 0.01, *** *p* < 0.001 (*p* value at category level).

p value: F test (post-regression ANOVA)

### Gender

Being a man was associated with a small but statistically significant increase in self-perceived competence in Model 1 (β = 0.07, CI: 0.02, 0.11), compared to being a woman. This effect diminished and became non-significant in the subsequent models.

### Occupation

Being a police officer, compared to being a healthcare worker, was significantly associated with high self-perceived competence across all models. However, in Model 3, the overall variable ‘Occupation’ did not show a significant effect on the service providers’ self-perceived competence (*p* value = 0.094). Within the categories, however, being a police officer demonstrated a significant positive relationship (β = 0.07, CI: 0.01, 0.14).

Being a social worker compared to being a healthcare worker was significantly associated with higher self-perceived competence in only two models: Model 2 (β = 0.08, CI: 0.04, 0.12), which adjusted for sufficient employer support for women with disabilities and gender, and Model 4 (β = 0.06, CI: 0.02, 0.11), which controlled for gender and the availability of routines to support people with disabilities exposed to IPV. There was no statistically significant difference for those in the ‘other’ occupational category compared to those working in the healthcare sector.

### Hours of training

There was a progressive increase in self-perceived competence among service providers as the hours of training focusing on IPV against women with disabilities increased. Specifically, the improvements were as follows: 17% for 1–5 hours (β = 0.17 CI: 0.13, 0.21), 18% for 6–10 hours (β = 0.18 CI: 0.11, 0.25), 27% for 11–20 hours (β = 0.27 CI: 0.18, 0.36), and 34% for more than 20 hours (β = 0.34 CI: 0.27, 0.41). Notably, service providers who reported more than 20 hours of training demonstrated the highest self-perceived competence, after adjusting for gender and occupation, as shown in Model 1.

### Sufficient employer support

After adjusting for gender and occupation, as shown in Model 2, both full and partial support from employers significantly enhanced self-perceived competence among service providers. Full support was associated with a 20% increase in self-perceived competence (β = 0.20 CI: 0.16, 0.25), while partial support corresponded to a 13% increase (β = 0.13 CI: 0.09, 0.17) compared to no employer support.

### Routines to support IPV victims

The availability of routines to support IPV victims positively influenced service providers’ self-perceived competence. Having such routines was associated with a 22% increase in self-perceived competence (β = 0.22 CI: 0.19, 0.26), compared to no routines, after controlling for gender and occupation in Model 3.

### Routines to support people with disabilities exposed to IPV

Model 4 shows that the availability of routines to support people with disabilities exposed to IPV was associated with a significant increase in self-perceived competence. After adjusting for gender and occupation, service providers who indicated having such routines showed an 18% increase in self-perceived competence (β = 0.18 CI: 0.15, 0.21) in comparison to those who had no such routines.

In Model 5, when all the variables were considered together, the number of training hours, employer support, and general routines to support IPV victims significantly influenced the service providers’ self-perceived competence. However, having specific routines for disabled IPV victims did not show a significant effect on service providers’ self-perceived competence when adjusted for all the study variables.

## Discussion

Our study’s findings show that professionals in healthcare, the police, and social services frequently encounter women with disabilities in their daily work, particularly those subjected to IPV. However, our data suggests that these professionals seldom ask directly about IPV when interacting with women with disabilities. A substantial proportion of participants did not state whether they routinely inquired about IPV. Although the service providers in this study stated that their institutions do have formal routines in place to support people with disabilities, victims of IPV, and specifically disabled victims of IPV, the actual utilization of these procedures appears inconsistent. Notably, many service providers mentioned that they had never utilized the specific routines designed to support disabled victims of IPV. We posit that the lack of responses regarding routine inquiries about IPV may reflect a general reluctance or limited experience in providing IPV support for women with disabilities. Moreover, the inconsistent use of the available routines suggests potential gaps in their implementation, which may limit the effectiveness of service providers in supporting IPV victims, particularly women with disabilities.

This study has assessed service providers’ self-perceptions of their competence in providing IPV support for women with disabilities. Specifically, we examined their ability to routinely inquire about IPV, recognize signs of abuse, manage IPV cases, provide intervention or support, and refer victims to appropriate services or institutions. Our results indicate that, to some extent, male service providers perceived themselves as more competent in providing IPV support compared to their female counterparts. Although data on this issue is limited, we argue that men in general may tend to overestimate their abilities. For instance, a Finnish study found that male dentists had higher self-confidence in performing most procedures compared to female dentists [26]. However, contrary to our study, a Swedish study in healthcare by Lawoko et al. showed that female providers demonstrated greater confidence, particularly in asking women about their exposure to IPV [27]. While Lawoko et al.’s study focused only on healthcare professionals, this study includes service providers from the police, healthcare, and social services sectors.

Our results also highlight that police officers report greater confidence in providing formal IPV support than other professional groups. Although there is limited research specifically on the police, this finding may reflect the structured reporting systems in police work and their role as a primary point of contact for physical abuse cases in particular. As we found in our previous qualitative study, IPV survivors reported that physical abuse is more straightforward to report to the police because the evidence is discernible [28], but also, like men, police officers may tend to be overconfident in their abilities, potentially boosting their confidence.

Training on IPV and disability emerged as a key factor influencing service providers’ perceived competence. Our data shows that more training hours directed towards supporting disabled victims of IPV significantly enhance service providers’ confidence in providing IPV support. Training is especially crucial to improve understanding of the needs of disabled IPV victims, as noted by Phillips et al. [20]. Given that women with disabilities are particularly vulnerable to violence, tailored training is recommended to equip service providers to meet their specific needs [29]. Similarly to our findings, previous Swedish studies have identified training as a key factor in ensuring that, for instance, social workers regularly inquire about IPV [22], and have emphasized the value of IPV-focused training in boosting service providers’ self-perceived competence and willingness to address IPV [18]. However, despite the recognized importance of training, many social workers reported feeling inadequately prepared to handle IPV cases [22]. Elsewhere, in a Spanish primary care setting, increasing the number of training hours was found to enhance professionals’ readiness to respond to IPV [30]

This study’s results also show that institutional support plays a critical role in shaping service providers’ self-perceived competence. We found that sufficient support from employers on how to support disabled victims of IPV, along with the availability of structured routines, was associated with increased confidence about providing IPV support. Specifically, routines designed for general IPV victims had a more substantial influence on service providers’ self-perceived competence than those tailored for disabled victims. Our descriptive data revealed that service providers with access to specific routines for disabled IPV victims rarely used them. This may reflect a lack of awareness, inadequate training, or low confidence in applying these specialized routines. Additionally, the regression analysis showed that the availability of routines for disabled victims had no significant effect on service providers’ self-perceived competence when controlling for other factors. Several potential reasons could explain these observations. Service providers may be more comfortable using general routines for IPV victims due to their broader applicability and greater familiarity. In contrast, routines for disabled victims may be seen as too specific or insufficiently adaptable to individual cases, leading providers to rely on the general ones, which they perceive as more effective. The low usage of disability-specific routines may also stem from the discomfort surrounding disability, which Freud describes as the ‘uncanny’ [31,32]. This discomfort may lead service providers to avoid using established guidelines and routines because disability can evoke a sense of unease – an unconscious experience of something unsettling, or strange. This psychological discomfort can make it difficult for service providers to offer effective support when women with disabilities present with IPV, even when reliable routines are available.

Prior research has emphasized that service providers equipped with clear protocols are essential for a targeted response to IPV, because this enhances their preparedness and increases the likelihood of inquiring about IPV [33]. Also, the service providers’ ability to prevent IPV, minimize its impact, and enforce laws is influenced by (in)adequate institutional capacity [34]. Similarly, a study in Sweden found that structured administrative procedures increased the likelihood of social workers routinely asking about IPV [22]. Our findings support these studies, showing that the availability of general routines boost service providers’ confidence. However, while general routines positively influence confidence, there is a need for improved awareness about and practical application of specialized routines for improving service providers’ self-perceived competence in supporting women with disabilities subjected to IPV.

## Methodological considerations

To minimize the risk of measurement errors, the survey questionnaire underwent a rigorous metrology standard review conducted by SCB’s measurement experts. This comprehensive analysis focused on the questionnaire’s structure, the wording of questions, and the response options to identify and correct any potential errors. The review also assessed the implications of these errors and provided recommendations for improvement. The entire process was documented in a detailed report submitted to the project’s principal investigator. Despite these efforts, our study has several limitations. Firstly, nuances in the questionnaire design – such as question wording and language translations – may have influenced how respondents interpreted and answered the questions, even after the metrology standard review. Another limitation is the relatively low response rate (32.9%), which may affect the generalizability of the findings. Additionally, the use of Yes/No response formats may not have fully captured the depth of service providers’ experiences or competencies, which could have contributed to a higher non-response rate. However, we deemed our sample size of 1,151 respondents sufficiently large to proceed with the analysis. Additionally, non-response bias may have influenced the results. Those who chose not to participate may differ systematically from those who did, possibly skewing the data in ways that are not immediately apparent. Finally, the survey relied on participants’ ability to recall events from up to one year previously, introducing the potential for recall bias.

## Conclusion

This study is, to the best of our knowledge, the first to assess the self-perceived competence of service providers from different formal support institutions. It shows that the service providers in these institutions frequently meet women with disabilities exposed to IPV, but rarely ask about IPV exposure. Furthermore, although the support institutions have routines in place to support disabled victims of IPV, the usage of such routines is low. In terms of the factors influencing service providers’ competence to optimally provide IPV support to women with disabilities in Sweden, our study suggests that professional training related to disabled IPV victims, along with adequate employer support for service providers meeting women with disabilities who are victims of IPV, positively influence those service providers’ confidence in their ability to effectively support women with disabilities subjected to IPV. However, service providers need targeted training and practical tools that focus on implementing specific routines to better meet the unique needs of disabled victims. Also, further research is needed to shed light on why the availability of specific routines for disabled IPV victims does not have any influence on service providers’ self-perceived competence.

## Data Availability

The datasets used and/or analyzed during the current study are available from the corresponding author on reasonable request.
